# Low Platinum-Content Electrocatalysts for Highly Sensitive Detection of Endogenously Released H_2_O_2_

**DOI:** 10.3390/bios12090672

**Published:** 2022-08-23

**Authors:** Ana Morais, Patrícia Rijo, Belen Batanero, Marisa Nicolai

**Affiliations:** 1 CBIOS—Universidade Lusófona´s Research Centre for Biosciences & Health Technologies, Campo Grande 376, 1749-024 Lisbon, Portugal; 2 Department of Organic Chemistry & Inorganic Chemistry, University of Alcala, 28805 Alcala de Henares, Spain; 3 iMed.Ulisboa—Research Institute for Medicines and Pharmaceutical Sciences, Faculty of Pharmacy, University of Lisbon, Av. Prof. Gama Pinto, 1649-003 Lisbon, Portugal

**Keywords:** hydrogen peroxide, electrochemical sensing, cathodic reduction, platinum-based electrocatalysts, bimetallic alloys

## Abstract

The commercial viability of electrochemical sensors requires high catalytic efficiency electrode materials. A sluggish reaction of the sensor’s primary target species will require a high overpotential and, consequently, an excessive load of catalyst material to be used. Therefore, it is essential to understand nanocatalysts’ fundamental structures and typical catalytic properties to choose the most efficient material according to the biosensor target species. Catalytic activities of Pt-based catalysts have been significantly improved over the decades. Thus, electrodes using platinum nanocatalysts have demonstrated high power densities, with Pt loading considerably reduced on the electrodes. The high surface-to-volume ratio, higher electron transfer rate, and the simple functionalisation process are the main reasons that transition metal NPs have gained much attention in constructing high-sensitivity sensors. This study has designed to describe and highlight the performances of the different Pt-based bimetallic nanoparticles and alloys as an enzyme-free catalytic material for the sensitive electrochemical detection of H_2_O_2_. The current analysis may provide a promising platform for the prospective construction of Pt-based electrodes and their affinity matrix.

## 1. Introduction

As a natural metabolite commonly found in all aerobic organisms [1], accurate surveillance of hydrogen peroxide (H_2_O_2_) is of significant interest in biomedical research, given its physiological and pathological roles.

This paper proposes various Pt-based alloys and composites as suitable catalytic electrode layers for the accurate and sensitive detection of H_2_O_2._ These Pt-based alloys take advantage of the embedded secondary metal (for instance, transition metal) nanoparticles’ synergistic effect and the increment of the effective area to enhance the sensing electrode’s signal.

Hydrogen peroxide (H_2_O_2_) is an existing non-radical oxidant in virtually all aerobic organisms. As one of the simplest peroxides that vary according to the medium’s pH, H_2_O_2_ can act as an oxidising or reducing agent [2]. Both redox reactions give rise to environmental-friendly side-reaction products, water or oxygen. Thus, for environmental reasons, H_2_O_2_ is preferred over other chemicals in most industrial applications.

H_2_O_2_ [3] is a covalent liquid, readily miscible with water, hence is a chemical compound that plays some pivotal roles in a wide range of industrial applications. These include the processing of petrochemicals (plastic industries) [4], paper-products manufacturing (pulp and paper bleaching) [5], textile industry [6], chemical industries [7], medical diagnostics [8], cosmetics [9], and biological analysis and biochemistry [10]. In addition, H_2_O_2_ acts as an essential mediator in environmental analysis, pollution control [11], pharmaceutical industries (research and manufacturing), and clinical research [8].

Given the inherent antiseptic and antibacterial properties of H_2_O_2_, it is also used as a cleaning product [12] and in food processing [13]. Therefore, H_2_O_2_ is commonly used as an antiseptic and sterilising agent in the food industry (e.g., sterilisation of aseptic packaging containers [14] to prolong products’ shelf life). Therefore, it is involved in cold pasteurisation and milk preservation to slow down the growth of bacteria and delay the fermentation of products [15].

In addition, as a powerful oxidising agent, H_2_O_2_ is widely used in the mining process [16]; it aids in synthesising various organic compounds [17] and works as an oxidant for constructing liquid-based fuel cells [18]. It is also employed in treating contaminated soils and wastewater, given its enrolment in Fenton’s reaction with the formation of the highly reactive hydroxyl radicals responsible for the aromatic and halogenated compounds’ withdrawal [19,20].

Biochemically [3], H_2_O_2_ is a ubiquitous small molecule of living cells that arises during the incomplete reduction of molecular oxygen as a by-product of cellular metabolism. It is thereby an uncharged molecule, which results as a side-product from a wide range of biological processes. Namely from a broad spectrum of selective oxidases in the mitochondria, such as glucose oxidase (GOx), cholesterol oxidase (ChoOx), glutamate oxidase (GlOx), oxalate oxidase (OxaOx), lactate oxidase (LOx), urate oxidase (UOx), alcohol oxidase (AlOx), lysine oxidase (LyOx), D-amino acid oxidase (DAAO), and xanthine oxidase, among others. H_2_O_2_ can also result as a direct product of superoxide dismutation by the superoxide dismutase enzyme (SOD) [21].

Because H_2_O_2_ is a prominent mediator product of various redox reactions, it is possible to monitor and establish the concentration of other biological species involved, such as glucose, lactate, urate, and cholesterol [22], by measuring the level of H_2_O_2_.

As a chemical substance, and given its relatively long lifetime at a physiological level, uncharged H_2_O_2_ can diffuse freely through phospholipid membranes, travelling within and between cells [23]. Therefore, H_2_O_2_ is allowed to enter other cell compartments’ readily reaching its cellular targets, which might induce various biological damages when found at oxidative stress levels.

Because of its ability to react with ubiquitous transition metal ions, H_2_O_2_ can readily generate one of the most reactive ROS, through Fenton or Haber–Weiss reactions, the hydroxyl radicals (OH^•^) [24]. Above physiological levels, H_2_O_2_ can be potentially toxic in the body after reacting with ferrous (Fe^2+^) or cuprous (Cu^+^) ions, frequently attainable at the active structural sites of proteins. The hydroxyl radical can indifferently react with all found molecules (lipids, proteins, nucleic acids, and carbohydrates), oxidising them. As a result, OH^•^ leads to severe oxidative damage in numerous molecules that comprise body tissues, with phospholipids, proteins, and DNA as the most targeted biomolecules in cell membranes [24].

Alongside the release of endogenous H_2_O_2_, there is an antioxidant system composed of several enzymes that balance the oxidative species’ concentration. Some enzymes designed to eliminate H_2_O_2_ at the cellular level, preventing its oxidative effects [25], are catalase, glutathione-peroxidases, and peroxiredoxins.

However, when there is a depletion of antioxidant species in the organism, an imbalance between the rate of the ROS endogenous production and the antioxidant mechanism that neutralises or repairs the resulting damage occurs [1].

For some time now, a significant amount of scientific evidence has pointed to oxidative stress’s prominent role in many pathological conditions [1,26]. The excessive presence of multiple distinct ROS and reactive nitrogen (RNS) species among the biological fluids disrupts cellular redox homeostasis, of which H_2_O_2_ is the most widely representative. As a result, the accumulation of H_2_O_2_ is closely correlated with an increased risk of autoimmune [27], inflammatory [27], and angiocardiopathic diseases [27], and likewise is involved in the own ageing natural process [28], including the age-related neurodegeneration [29]. Therefore, H_2_O_2_ is an early indicator of ischemia/reperfusion injury [1], traumatic brain injury, neurodegenerative disorders, impaired learning and memory functions [28], atherosclerosis, myocardial infarction [27], diabetes, cancer [24,30], and related kidney injury diseases [31], and others.

Apart from the adverse effects stated above, in terms of the vital physiological functions of living organisms, H_2_O_2_ is an essential metabolite that, thanks to its free transit across cells, acts as a mediator in most cellular metabolic reactions [26]. Therefore, H_2_O_2_ is involved in many biological processes with a significant role at the cellular level of the human organism. Accordingly, it participates in the host-defence mechanism of the immune system and as a signalling molecule in the regulation of diverse biological processes such as the coordination of protein synthesis, immune-cells activation, vascular remodelling, and cell apoptosis.

In eukaryotes, particularly in higher vertebrates, endogenous H_2_O_2_ mediates several physiological processes [29]. It is therefore generated in precise locations close to the responsive signalling molecules. Additionally, H_2_O_2_ may be evenly produced by activating NADPH oxidases in target cells. Therefore, H_2_O_2_ act as a secondary messenger in multicellular organisms, emerging as a specific response to growth factors and cytokines in cell division, differentiation, and migration [32]. Hence, H_2_O_2_ is involved in modulation and signalling redox metabolism under physiological conditions as a signalling molecule. It regulates cell growth, proliferation, and differentiation and is further responsible for cellular migration.

As a second messenger, H_2_O_2_ triggers a series of specific oxidations (through the oxidative modulation of sensitive redox-proteins, also called redox switches), which activates the proteins’ sequence downstream as the cell’s metabolic response. Occurrence, which in turn enables cellular’ proliferation, survival, or extinction.

The outcome depends on which downstream pathways have been activated: homeostatic, pathological, or protective. Hence, when at low physiological levels, H_2_O_2_ can also initiate protective responses to limit or repair cellular oxidative damage [1,33].

The disposal of metabolites, such as H_2_O_2_, represents a metabolic mechanism that allows regulating their levels in the human body. Since the ubiquitous H_2_O_2_ is usually excreted through body fluids such as urine, tears, and sweat or in breathing during the regular functioning of the human body, the concentration of H_2_O_2_ found in those samples can be a valuable tool to assess the degree of oxidative stress on the human organism [10]. Therefore, H_2_O_2_ works as a biomarker, a parameter used to diagnose and survey various diseases.

## 2. Hydrogen Peroxide Electrochemical Detection

Within the sphere of development of biosensor technologies for medical applications aiming to surveil a myriad of disorders, the accurate quantification of metabolites involved in the most common persistent health conditions has increasingly attracted attention. Particularly H_2_O_2_, due to its role as a metabolite implicated in a broad spectrum of different health disorders [31,34,35,36] and its central role as a regulator of various biological processes. As a result, a high-sensitive quantification of H_2_O_2_ is relevant for developing a reliable diagnosis of the different medical conditions in humans and, hence, subject to considerable research efforts of strict detection requirements.

### 2.1. H_2_O_2_ Sensing Analytical Methods

The scope of different analytical methods used in the determination of H_2_O_2_ has included the techniques: fluorimetry, spectrophotometry, fluorescence [37], chromatography [38], chemiluminescence [39], titrimetry [40], and electrochemistry [41]. However, most of these methods reveal some inherent technical drawbacks, such as low sensitivity and selectivity, long testing time, vulnerability to interferences, and complicated or expensive equipment. Among the existing techniques for H_2_O_2_ detection, the electrochemistry approach has proved to be the most attractive/efficient tool [41]. It allows for a short-term accurate and selective evaluation of a low detection limit on low-cost instrumentation. Moreover, it is one of the most versatile methods because it holds a wide dynamic span, simple instrumentation, and easy miniaturisation, becoming suitable for quantifying changes in H_2_O_2_ levels in vivo in the brain [42]. Furthermore, electrochemical technology based on the catalytic activity allows for an H_2_O_2_ detection method of simple, fast, and cost-effective operation [43] once H_2_O_2_ is an electroactive molecule that can be oxidised or reduced on the surface of a solid electrode.

Nevertheless, the electrochemical processes might be constrained by the sluggish kinetics of the electrodes, and the high overpotential required [44], which will reduce the sensing performance. Consequently, the electrode’s catalytic activity for H_2_O_2_ detection is crucial in the sensor/biosensor device’s performance. Therefore, modification of the electrodes is an essential task for developing a reliable sensor/biosensor.

### 2.2. H_2_O_2_ Electrochemical Detection by Enzyme-Based Modified Electrodes

Considering that H_2_O_2_ is a product of some oxidases’ catalytic activity [26], biosensing of H_2_O_2_, based on its electrochemical oxidation/reduction, is frequently favoured by the presence of some specific biological molecules with high peroxidase activity [45]. Accordingly, H_2_O_2_ biosensing is commonly performed using a protein/enzyme-based modified electrode, depending on the biomolecule supported. Monitoring H_2_O_2_ by its redox reaction becomes faster, more sensitive, and selective when performed in certain heme-proteins such as haemoglobin or other natural enzymes such as the horseradish peroxidase and heme-containing enzymes, such as cytochrome-c peroxidase [45].

Moreover, given that H_2_O_2_ is a bioactive molecule, resulting as a by-product of numerous enzyme-catalysed reactions, it renders H_2_O_2_ a “reporter” molecule that might be used to identify other biomolecules, thus enabling the electrochemical detection of other non-electroactive metabolites [46]. Consequently, considerable attention has been given to the research and design of new, more sensitive electrodes that address H_2_O_2_ detection and improve the detection of non-electroactive species labelled by H_2_O_2_ quantification. In this scope, the detection of H_2_O_2_ by enzyme-based biosensors is of great importance, as H_2_O_2_ provides the indirect detection of many non-electroactive targets otherwise implausible to be measured. The glutamate and adenosine neurotransmitters are some inactive targets [47,48]. Specific oxidases are immobilised onto the electrode surface, converting the non-electroactive biological target to H_2_O_2_ for the subsequent in vitro detection of the attendant targeted molecule. This type of screening has been mainly used in the enzymatic detection of glucose by involving glucose oxidase (GOx) assistance in biosensing due to their crucial role in controlling glucose in diabetic patients [49].

However, certain constraints are related to the use of biological material for the analytical detection of H_2_O_2_. Namely, the not-always easy process of enzyme-immobilisation [50], the requirement of some molecules on modified electrodes, a mediator between target species and the electrode surface, and the choice of material used as a matrix to restrain molecules on the electrode [51].

Although enzyme-based detection is still a dominant bioanalytical process, this procedure has several shortcomings, such as its complexity, lack of stability, and high cost [52]. This hindrance happens because the enzymatic activity is affected by temperature, pH, atmospheric O_2_ and the enzyme’s intrinsic nature, leading to enzymatic degeneration, resulting in poor stability and low reproducibility of enzymatic biosensors. Considering all the previously reported issues, developing enzyme-free H_2_O_2_ sensors has become highly desirable.

### 2.3. H_2_O_2_ Electrochemical Detection by Metallic Nanoparticles-Based Electrodes

The development of different modified electrodes for the detection of H_2_O_2_ has attracted a great deal of attention since the selectivity of the electrochemical response to H_2_O_2_ occurrence is improved by using various components catalytically active in this modification. Numerous modified electrodes without any enzymes or other proteins immobilisation have already been reported for the electrochemical detection of H_2_O_2_ [53]. In particular, metallic-based electrodes have shown excellent catalytic activity towards both H_2_O_2_ redox reactions [54]. Under this scope, over recent years, research has been performed [55] to evaluate the most suitable materials to apply on the electrode surface to attain the most proficient electrochemical detection of H_2_O_2_.

Current research regarding the electrochemical detection of H_2_O_2_ focuses on the survey for the most efficient electrocatalytic materials, aiming for H_2_O_2_ direct sensing.

Recent development in nanoscience has brought the proliferation of nanomaterials with essential roles in several areas such as catalysis, sensors, biomedicine, biological labelling, surface-enhanced Raman scattering, and microelectronics [56]. This remarkable growth in nanomaterial research was mainly due to these materials’ unique chemical, physical, and electronic properties at their nanoscale. In practice, the difference in the solid materials’ properties according to their scale stems from the valence band and conduction band’s energy levels gap, which are involved in the chemical bonding of the several atoms composing their structure. As the material’s size grows nearer to the electron transfer free path, electrons’ transfer between the atoms that constitute that material becomes more unlikely as the d-bands are further apart. Thus, unlike bulk material, the nanomaterial features discrete energy levels due to the constraint of the electrons’ wave-function caused by their limited physical dimensions. Accordingly, these unique characteristics are reflected in the exceptional electrical, thermal, catalytic, and optical properties that differ from those of the same bulk materials. On the other hand, nano-sized structures efficiently expose their high specific electrochemical active surface area (ECSA) to connect with the substrate molecules.

The metallic nanoparticles exhibit exceptional capabilities in the catalysis field, mainly due to their high surface-volume ratio and high crystallinity level, which provides the necessary conditions for higher catalytic performance. This property is responsible for the increased number of active sites for electron transfer, making the catalytic approach more efficient. Therefore, metallic nanoparticles are widely used for direct electron transfer in sensor and biosensor devices [57]. The adoption of new surface analysis approaches and the feasibility of structural relationships’ modelling of desired materials has enabled the construction of advanced electrodes with tailor-made metal nanoparticles [58].

A reliable catalyst consists of a material that reduces the overpotential required for the electrochemical reaction and improves the kinetics of its electron transfer. In this context, carbon-based materials have revealed high catalytic activity for oxidation and reduction of H_2_O_2_. Some of the most frequently used carbonaceous [59] are graphite, glassy carbon, reduced graphene oxide, carbon nanotubes, and carbon paste electrodes.

Simultaneously, metallic nanoparticles on modified electrodes increase the electrocatalytic activity for H_2_O_2_ detection since they significantly improve the electrode signal [60]. One of the common characteristics of metals is their susceptibility to donate and accept electrons from their valence orbital, readily submitting to the redox process. Specifically, transition metals tend to acquire multiple valence states so that they undergo dynamic transitions between these valence states. This remarkable ability renders transition metals a type of material able to operate as an electron-carrier species during oxidation–reduction reactions.

The transition metal nanoparticles’ properties induce rapid mass transport and electron transfer during the heterogeneous electrocatalytic process, causing catalysis at the electrode surface to be very effective. Therefore, the signal amplitude of a nano-metallic composite electrode might result from a concerted action between the atomic structure of the metals and the nano-sized material’s specific properties. Due to its relative-inert nature, noble metal nanoparticles are preferred over other transition metal compounds to construct catalytic materials. Considering d-subshell electrons’ higher exposure, the dissociative adsorption of adsorbent species on its surface becomes more expedited.

Among the most recently studied metallic nanoparticles, platinum nanoparticles (Pt NPs), single or mixed with other species, disclose a high catalytic effect on the oxygen reduction reaction. Specifically, in heterogeneous catalysis, platinum nanomaterials have been comprehensively analysed as promising materials since their’ porosity additional feature enhances their electrochemical active surface area and consequently sustains the electron transport capacity [61].

Over the last 30 years, platinum nanoparticles (Pt NPs) have been extensively studied in electrochemical applications [62], given their high electrocatalytic efficiency and selectivity. Since mid-2015, Pt NPs-based electrodes have been widely used in the enzyme-free sensing of H_2_O_2_ [63] due to their high electrocatalytic activity and selectivity for H_2_O_2_. Most Pt NPs-based non-enzymatic sensors exhibit an excellent response to H_2_O_2_ with a wide linear range, low detection limit, and good stability. In addition, the detection of H_2_O_2_ at low potential will lead to good selectivity.

Although noble metals exhibit good catalytic performance, their high cost and poison susceptibility [64] limit their potential for large-scale applications and emphasise the demand for alternative, less expensive and readily available high-efficiency catalytic materials. One of the biggest threats to platinum catalytic performance is CO contamination during heterogeneous catalysis. This interference results from the molecular structure of CO, whose triple bond justifies the strong binding capacity on the platinum surface, hindering the analyte from accessing the active catalytic sites available for heterogeneous catalysis [64]. However, the platinum electrocatalyst’s tolerance to carbon monoxide poisoning can be improved by doping the platinum with other materials such as transition metals [65], oxides or high-surface-area carbonaceous such as multi-walled carbon nanotubes [66]. The incorporation of a different transition metal in the platinum surface structure contributes to the attenuation of CO poisoning due to the alteration of the local electronic structure of platinum at the reaction active site. The resulting hybridisation between the d orbitals of the transition atoms in the alloy reduces the energy of platinum d- orbitals contributing to the rise in CO binding energy, weakening it. Moreover, under the inclusion of a highly oxophilic transition metal (high binding affinity for oxygen), the additional bifunctional effect contributes to CO poisoning surpass. Since at a lower potential, it can oxidise water molecules into hydroxyl species (OH), which react with the CO adsorbed on the adjacent Pt sites, forming CO_2_ and H_2_ that are released, regenerating the active sites of Pt [67]. On the other hand, CO poison adsorption at the Pt surface was also observed by the modification of Pt electrodes with metallic oxides or carbonaceous that, while increasing the dispersion of PtNPs in the composite structure, breaking the required contiguity of Pt active sites for the adsorption of CO [66].

Since the early first decade of the 21st century [41], many Pt-based bimetallic alloy nanoparticles have been suggested to construct H_2_O_2_ electrochemical sensors.

In general, Pt NPs exhibit good electrocatalytic behaviour towards the H_2_O_2_ electrochemical reduction. Likewise, they have been widely used at negative potentials, i.e., under cathodic polarisation conditions, preventing possible interferences of the coexisting electroactive species, such as ascorbic acid (AA) and uric acid (UA) [68]. Nevertheless, despite the catalytic activity toward H_2_O_2_ reduction reaction, some Pt NPs-modified electrodes might face an interference of dissolved O_2_ under cathodic conditions_,_ which is difficult to overcome due to its highly negative reduction potential [69,70].

The introduction of an extra atom to the solid platinum structure usually shifts the electrical potential to more negative values, which most often aids in avoiding the possible interferences from concomitant species in the analyte solution [70,71].

**Figure 1:**
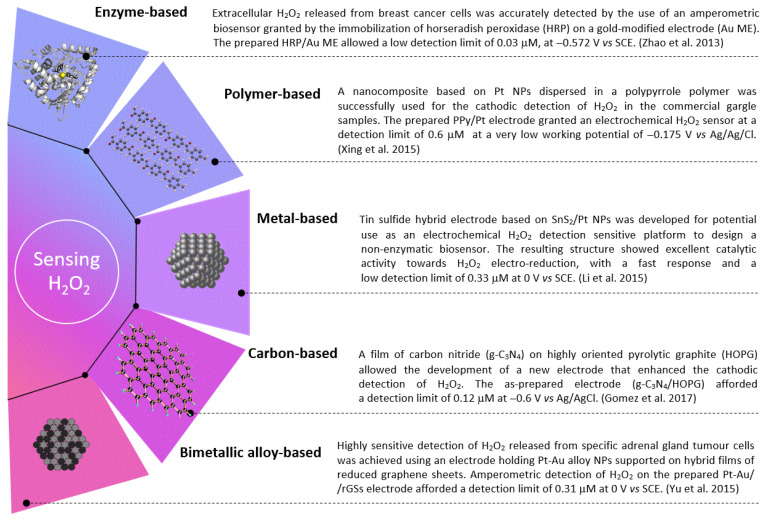
The different electrodes commonly used for the sensitive detection of H_2_O_2_. Collected data from the research works of Zhao et al. [35], Xing et al. [86], Li et al. [63], Gomez et al. [59], and Yu et al. [69].

Whenever possible, adjustments to the electrode are desirable by incorporating several materials to increase the catalytic effects resulting from such materials’ synergy. Some materials have shown distinct advantages over conventional materials for H_2_O_2_ detection when combined, adapted, or rearranged. Several H_2_O_2_ sensing’ electrodes are illustrated in the graphical diagram from Figure 1.

By setting a recognition element of a new sensor, the new electrode is designed by assembling a structure composed of several different compounds, nanoparticles, oxides, and metals collected to outline a new platform on the surface of the electrode. The new electrode platform is accomplished by supporting the electrocatalyst on the electrode surface.

#### 2.3.1. Pt-Based Nanocomposite Structures

Pt-based nanocomposite systems with NPs of different structures (alloyed NPs, mixed monometallic NPs, or core–shell NPs) have proved to be novel architectures with remarkably improved catalytic activity. Therefore, they are used extensively as electrodes for methanol oxidation and oxygen reduction reactions in fuel cell technology [72]. Moreover, bimetallic alloys in sensor development have also been progressing by taking advantage of synergistic effects resulting from metals used in the alloy [73].

For instance, Niu et al. [74] proposed the formation of some nanoclusters with excellent catalytic performance for the reduction of H_2_O_2_ by the deposition of Pt-Pd nano-agglomerates on gold nanofilm substrate (SPGFE/Pt-PdBNC). The prepared 3D electrode showed a higher current response concerning the H_2_O_2_ electrochemical reduction in a neutral environment than the monometallic Pt and Pd nanomaterials. After successive addition of H_2_O_2_ in 0.1 M PBS (pH 7.0), the amperometric results showed that the current values increase linearly with H_2_O_2_ concentrations ranging from 0.005 to 6 mM. The snowflake-shaped Pt-Pd alloy nanostructure carries more catalytic sites for H_2_O_2_ reduction, thus making it more sensitive to the addition of low concentrations of H_2_O_2_ at 0.4 V vs. Ag/AgCl at the 804 μA mM^−1^ cm^−2^ measurement.

Mei et al. [75] have prepared a novel sensor based on Fe@Pt/C core–shell core–shell nanocomposites with excellent catalytic activity toward H_2_O_2_ reduction. The Fe@Pt core–shell structures with a thin Pt shell layer on the non-noble metal core were synthesised by a sequential reduction process and subsequent Fe@Pt/C by the dispersion of bimetallic NPs on Vulcan XC-72 carbon. The voltammetric results suggest that the Fe@Pt/C provided the necessary conduction pathways to promote the electron transfer rate and the Fe@Pt NPs-modified electrode has a wide ECSA. The new sensor based on Fe@Pt/C nanocomposites exhibited an excellent catalytic activity for reducing H_2_O_2_ at a working potential of −0.4 V vs. saturated calomel electrode (SCE), emphasising its practical utility for H_2_O_2_ sensing. The results have shown that the Fe@Pt/C electrode displays good catalytic activity for H_2_O_2_ with high sensitivity, good stability and excellent reproducibility. Chronoamperometric measurements showed that the Fe@Pt/C-modified electrode exhibits a sensitivity of 218.97 μA mM^−1^ cm^−2^ and a detection limit of 750 nM from the linear relationship between the reduction current-signal and the concentration of H_2_O_2_ in the range of 25 μM to 41.605 mM. The applicability of the developed sensor was evaluated by verifying the amount of H_2_O_2_ in an antibacterial lotion (3% H_2_O_2_). The results are in line with the expected value; thus, the excellent catalytic performance of Fe@Pt/C makes it a promising application for H_2_O_2_ quantification. The improved catalytic performance of Fe@Pt [75] results from the electronic and structural effects [76] between a metal core and the shell of bimetallic core–shell NPs. This phenomenon can be ascribed to electronic effects since the electronegativity of Fe is lower than that of Pt, thus altering the electronic properties of Pt when the metals are adjacent as Fe@Pt-skin. Thus, nearby Fe, the density of the Pt d-band is reduced to lower energy [77], leading to a change in the chemical adsorption energies. Moreover, the induced modification of the electronic structure of Pt-skin by Fe can increase the number of active sites for analyte adsorption [77].

In addition, the group Zhao et al. [68] have prepared a high porous composite of core–shell Cu@Pt/C nanoparticles structure. Such an open-cell organisation with irregular pores has allowed them to provide a higher number of active sites to adsorb the analyte H_2_O_2_ and thus improve their electrocatalytic activity. The analysis performed by CV and XRD techniques shows that the improved electrocatalytic performance associated with Cu@Pt NPs is due to the structural and electronic modification of the Pt atoms near the surface/shell layer caused by the Cu core. X-ray diffraction (XRD) analysis shows that the Pt at the bimetallic core–shell particle surface exhibits lower binding energy in Cu@Pt NPs than the bulk Pt. The electrochemical survey showed that the currents obtained for H_2_O_2_ reduction increase gradually with increasing H_2_O_2_ concentration, indicating that Cu@Pt/C/GCE can be used to quantify H_2_O_2_ concentration. Compared to Pt/C, Cu@Pt/C has revealed better electrocatalytic activity for H_2_O_2_ reduction, showing a sensitivity of 351.3 μA mM^−1^ cm^−2^ and a limit of detection of 0.15 μM over a wide linear range of H_2_O_2_.

#### 2.3.2. Pt-Based@ Carbonaceous Structures

Carbonaceous is one of the most suitable materials to support the catalysts for a high-sensitivity sensor due to its characteristics, such as large surface area, porous structure, and good electrical conductivity [78]. Carbon-based materials are essential as they influence the kinetics and mechanism of the redox reaction at the electrodes. Generally, the kinetics of the electron transfer process in carbon-supported electrodes relies on the carbon structure and its surface treatment. However, the highly hydrophobic nature of carbonaceous makes them suitable for a superior catalytic activity in biosensing applications. Carbonaceous are low-cost support materials whose availability and simple removal from the electrode surface make them a favourable support platform for electrochemical measurements. Additionally, carbon-based electrodes have a high chemical and electrochemical stability over an extensive potential range in the negative and positive directions.

Zhao et al. [79] have developed a modified electrode by the electrodeposition of Pt NPs in flowers shape on Fe oxide supported on reduced graphene oxide (Fe_3_O_4_/rGO). Such electrocatalyst has proven to be a reliable and effective tool for H_2_O_2_ detection. The experimental CV shows that the current intensity on Pt/Fe_3_O_4_/rGO/GCE increased with H_2_O_2_ in 0.1 M PBS (pH 7.4), highlighting the rapid electron transfer at the electrode interface during the H_2_O_2_ reduction. From the impedance assays was also possible to check that the electrical resistance decreased as the various electrode sub-layers increased, from GCE > Fe_3_O_4_/GCE > Fe_3_O_4_/rGO/GCE > PtFe_3_O_4_/rGO/GCE. It suggests that graphene and Pt nanoparticles may be at the origin of the effective promotion of electron transfer. The amperometric characterisation of Pt/Fe_3_O_4_/rGO showed a sensitivity of 6.875 μA mM^−1^ from the linear range between 0.1 and 2.4 mM concentration of H_2_O_2_ and a detection limit of 1.58 μM. Furthermore, the Pt/Fe_3_O_4_/rGO sensor’s behaviour showed a favourable anti-interference characteristic on the coexisting species in biological samples. The electrochemical analysis results suggested that the proposed Pt/Fe_3_O_4_/rGO electrode could be an effective tool for the quantitative electro-cathodic surveillance of H_2_O_2_.

Several electrocatalysts with distinct degrees of N-graphene (N-Gr) loading were assembled and used as advanced electrodes for H_2_O_2_ detection by Tajabadi et al. [80]. The electrode was prepared by electrophoresis of N-graphene nano-sheets on indium tin oxide glass (ITO) substrate, on which nanoflower shaped (NF) Pt nanoparticles were electrochemically deposited. The PtNF-N-Gr has shown a fast amperometric response over a wide linear range of H_2_O_2_ (1 μM to 1 mM), denoting a high catalytic behaviour of the modified electrode towards H_2_O_2′_ reduction at the working potential of −0.40 V vs. Ag/AgCl. The amperometric response of the 0.05 mg ml^−1^ of N-graphene loading modified ITO electrode in nitrogen-saturated 0.1 M PBS solution to successive additions of H_2_O_2_ was used to explore the performance of the as-prepared electrochemical sensor. The hybrid electrode exhibited fast, stable, and reproducible current responses and a linear current response to successive additions of H_2_O_2_ in the PBS solution. This modified electrode showed the best catalytic performance with a detection limit of 0.34 μM. Electrochemical impedance spectroscopy analysis (EIS) also corroborated the higher catalytic ability of the electrode employing the charge of 0.05 mg ml^−1^ in N-graphene. This conductivity increment improves the electrocatalyst catalytic capacity, which results in a faster electron transfer process across the interface and better catalytic performance of the electrode when used as a sensor.

#### 2.3.3. Pt-Based@ Highly Porous Structures

The specific surface area of porous architectures relies on the size of their ligaments, their pores, and solid bulk densities [81]. Effectively, the porous material of the electrode has a high ECSA, also known as specific surface area. Moreover, the specific surface area of the porous architecture rises with the number of porous cages. The same goes for rough surfaces. The electrode roughness is the ratio between the electroactive surface area of the electrode and its geometric area. Usually, the roughness factor of nano-sized catalytic material is high as the electrode’s specific surface area is much higher than the geometric one. Porous and highly rough electrocatalysts promote heterogeneous electron transfer at the analyte/electrode interface. They provide a wider surface area to react with the analyte and increase the heterogeneous electron transfer. The unique catalytic activity of the platinum surface coupled with the high surface area granted by its roughness provides additional sites for the electrocatalytic reduction of hydrogen peroxide, thereby increasing the reaction rate on the electrode surface.

Effectively, Guo et al. [82] have prepared an electrode based on the coaxial nano-cables, which can readily adsorb high-density metal nanomaterials through the functionalisation with an NH_2_ group. Thus, this group formulated an electrode modified with a high roughness CNT/SiO_2_/(Au/Pt) nanostructure that showed improved catalytic skills towards the direct reduction of H_2_O_2_ at −0.15 V vs. Ag/AgCl, reaching the maximum cathodic constant current within 3.5 s. This swift performance was ascribed to the fact that H_2_O_2_ is readily diffused within the high ECSA of the CNT/SiO_2_/(Au/Pt) where it will react. The CV study verified that H_2_O_2_ cathodic peak currents increased with both concentration of H_2_O_2_ in 0.1 M PBS (pH 7.0) and scan rate from 0.02 to 0.2 V s^−1^. The amperometric analysis also disclosed a sensitive linear response of the modified electrode towards the H_2_O_2_ electroreduction from 0.5 µM to 1.67 mM, with a detection limit of ca. 0.3 µM. Moreover, such ultra-high-density Au/Pt NPs modified electrodes showed a high performance in the electro-oxidation of oxygen, methanol, and hydrazine.

A sensitive and selective amperometric detection of H_2_O_2_ in biological samples was accomplished by a new electrode prepared by Thirumalraj et al. [83] through the electrochemical deposition of Pt NPs on a mixture of graphite(Gr) and gelatin (GLN) protein. The combination of the high catalytic performance of graphite with the excellent matrix of the protein molecules comprising the gelatin makes the resulting hydrogel into a perfect immobilising platform for various biomolecules detection. The modified electrode PtNPs@GR/GLN showed an excellent electrochemical behaviour over H_2_O_2_ sensing with high electrocatalytic activity at a lower working potential for H_2_O_2_ reduction. Therefore it allowed the development of a non-enzymatic sensor for the selective detection of H_2_O_2_ in the presence of other biologically active interfering molecules such as AA, DA, and UA at a low detection limit of 37 nM. The superior dispersing action of GNL on high catalytic GR layers increases the number of active sites available in the electrode matrix. As a result, it fosters a heterogeneous electron transfer process at the interfaces of electrode/electrolyte. PtNPs@GR/GLN was successfully applied to electrochemical monitoring of H_2_O_2_ in biological samples of human serum and saliva.

#### 2.3.4. Pt-Based@ Conductive Polymer Structures

Conductive polymers are π-π-conjugated polymers [84] containing functional groups that allow the easy immobilisation of the recognition molecules. In general, conductive polymers have unique features, such as high electrical conductivity resulting from the delocalised π-electrons within the polymeric chain backbone and low ionisation potential. Commonly, metallic nanoparticles or metal oxides can be immobilised on the polymer structure, resulting in stable hybrid films of high catalytic activity. These large surface areas, high conductivity, and reduced charge/mass transport pathways sustain the potential success of conductive polymers as catalyst supports for electrochemical sensing devices.

Within the energy field investigation, Oliveira et al. [85] have prepared several polypyrrole/platinum composites tested for the cathodic reaction of H_2_O_2_ in acidic media. Pt-based assembled electrocatalysts supported conductive polypyrrole-carbon composite (PPy-C) containing different amounts of carbon black (5, 12, 20, 35%). Among the four electrocatalysts, Pt/PPy-C_35%_ exhibited the best electrochemical response toward the H_2_O_2_ cathodic reaction, showing a remarkable current density peak of 11.6 mA upon the addition of H_2_O_2_ to the supporting electrolyte at a relatively low potential of 0.8 V vs. RHE. The improved catalytic performance of Pt/PPy-C_35%_ for H_2_O_2_ cathodic reaction was sustained by the lower activation energy value than the remaining catalysts.

In the scope of non-enzymatic H_2_O_2_ detection, catalysts prepared and evaluated by various researchers have also been assessed as new sensors for H_2_O_2_ in different commercial samples [75] and at a cellular level [83].

For example, Xing et al. [86] have developed an electrochemical H_2_O_2_ sensor made of a polypyrrole/platinum composite, with platinum nanoparticles densely dispersed within those of PPy. This PPy/Pt electrocatalyst evidenced good catalytic activity towards H_2_O_2_ reduction, with an enhanced detection sensitivity at a lower working potential (c.a. −0.175 V vs. Ag/AgCl). Indeed, at this low working potential of −0.175 V, no significant current response was observed owing to the reduction of the coexisting O_2_, not affecting the electrochemical detection of H_2_O_2_. The sensor calibration curve shows a sensitivity of 305.45 μA mM^−1^ cm^−2^ over the linear range of 25 to 500 μM H_2_O_2_ and a detection limit of 0.6 μM. The PPy/Pt/GCE sensor used to construct a non-enzymatic electrochemical H_2_O_2_ detector showed superior sensitivity, selectivity, and accuracy. It was also successfully applied to detect H_2_O_2_ in commercial gargle samples, proving its feasibility in practical analysis.

#### 2.3.5. Pt-Based Bimetallic Alloys

The second atom within the electrocatalyst provides a vital strategic variation in its shape, size, and morphology, resulting in the amendment of its chemical and physical properties. Effectively, adding a second element to the metallic structure provides the synergistic effect of both materials, along with a cost reduction in the electrocatalyst due to the decline in the amount of most expensive metal, e.g., platinum [64]. Compared with the corresponding mono-metal NPs, bimetallic blends reveal higher catalytic activity, more reliable resistance to deactivation, and excellent catalytic selectivity.

Over the past two decades, several research groups have successfully developed different catalytic materials [41,73] based on Pt and transition-metal alloys targeting the selective detection of H_2_O_2_. Table 1 summarises the main analytical parameters of H_2_O_2_ detection by different Pt-based electrocatalysts developed by several researchers.

Mei et al. [87] have reported the development of a high selective enzyme-free H_2_O_2_ sensor based on PtNi bimetallic alloy onto multi-walled carbon nanotubes (MWCNTs). It exhibited excellent sensitivity, stability, and reproducibility, making it suitable for detecting H_2_O_2_ in real samples. The well-dispersed MWCNT granted a higher ECSA for PtNi active species, resulting in a highly electroactive hybrid composite. The improved electrochemical activity of PtNi/MWCNTs is ascribed to the mentioned higher ECSA. Voltammetric measurements performed in Mei’s work on the modified PtNi/MWCNT electrode pointed to a diffusion-controlled mechanism for the H_2_O_2_ reduction reaction on its surface. In practice, PtNi/MWCNTs electrode has revealed higher electrocatalytic activity towards H_2_O_2_ reduction, along with a unique sensitivity and selectivity for sensing H_2_O_2_ at the −0.45 V vs. SCE as working potential and pH 7.0. Accordingly, the prepared electrode accomplished a sensitivity of 2123.1 µA mM^−1^ cm^−2^ with a detection limit of 0.06 μM.

Similarly, Gutierrez et al. [88] have prepared a copper-rich core–shell structure decorated by a PtPd alloy shell supported on a glassy carbon electrode, which revealed substantial electrocatalytic activity for H_2_O_2_ reduction. The comparison of the modified electrode sensitivities by every single metal separately and by the PtPd alloy around the Cu nanostructure (Cu@PtPd/C) highlights the synergy of the different metals in the final composite for the reduction of H_2_O_2_ at −0.1 V vs. Ag/AgCl. It also shows a high sensitivity of 530.0 μA mM^−1^ cm^−2^ and a detection limit of 0.37 μM. The electrocatalyst was used to assemble a sensing platform that was successfully exploited to quantify H_2_O_2_ in a commercial mouthwash sample. The Cu@PtPd/C structure also shows excellent amperometric durability and long-term stability with good anti-interference for AA and UA in amperometric detection of H_2_O_2_ at −0.1 V. Furthermore, the voltammetric profiles obtained for 0.050 M H_2_O_2_ on GCPE paste and 5% *w*/*w* Cu@PtPd/C GCPE portray that core–shell NPs decrease both the oxidation and reduction potentials for H_2_O_2_. In addition, the significant improvement of the associated currents for both processes highlights the electrocatalytic effect of Cu@PdPt/C for H_2_O_2_ electrochemical redox reactions.

Under an energy supply-based approach, Morais et al. [18] have prepared the bimetallic electrodes of PtNi and PtCo NPs by immobilising them on a glassy carbon electrode (GCE) surface with a Nafion solution binder. The resulting Pt_0.75_M_0.25_/Vulcan XC-72 carbon black electrodes showed significant catalytic performance in the electrochemical reduction of 0.03 M H_2_O_2_, exhibiting current signals in the milliampere range. Similarly, the prepared Pt/C, Pt_0.75_Ni_0.25_/C and Pt_0.75_Co_0.25_/C electrocatalysts exhibited a rate constant for the cathodic reaction of H_2_O_2_ of 7.4 × 10^3^, 11.2 × 10^3^ and 11.8 × 10^3^ mol^−1^ cm^3^ s^−1^, respectively, showing a high catalytic performance for H_2_O_2_ reduction. Therefore, the tested catalysts should also exhibit high electrocatalytic performance and offer an excellent application as sensitive sensors for the physiological estimation of H_2_O_2_. In this study [18], the kinetic results showed a superior catalytic activity of carbon-supported Pt alloys for the H_2_O_2_ electrochemical reduction reaction compared to monometallic Pt. Accordingly, the dependence of catalytic activity on the nature of the alloying component has been shown so that, for instance, catalytic activity increased in the following order: Pt_0.75_Co_0.25_/C > Pt_0.75_Ni_0.25_/C > Pt/C.

Similarly, Cardoso et al. [89] have investigated different rare earth and platinum alloys (Sm, Dy, Ho) in an alkaline medium. The cyclic voltammetry analysis (CV) for 0.05 M H_2_O_2_ in 2 M NaOH solution at −0.1 V vs. reference hydrogen electrode (RHE) showed a higher current signal for the Pt-Sm alloy. There is strong evidence that these current density values attained may be related to the significant roughness of the Pt-Sm alloy surface and the increased active sites for the catalytic reaction.

**Figure 2:**
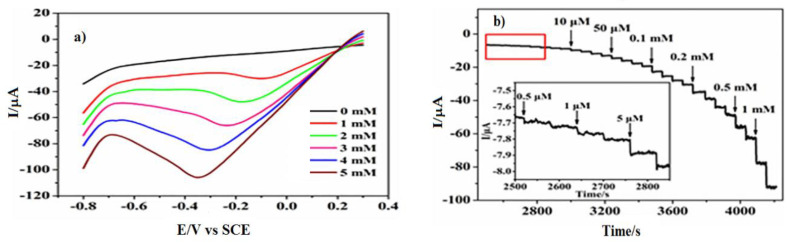

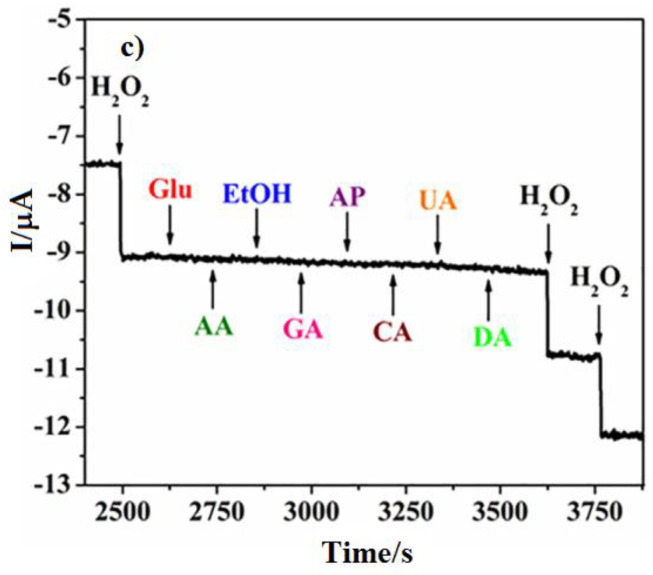
(**a**) Linear Sweep Voltammetry of the PtNi/NCNFs (3:1)-GCE electrode at different H_2_O_2_ concentrations. (**a**) Current-time response of PtNi/NCNFs (3:1)-GCE to the successive additions of H_2_O_2_ at −0.1 V. (**b**) Amperometric response curve at PtNi/NCNFs (3:1)-GCE to successive additions of 0.1 mM H_2_O_2_ and other electroactive species at −0.1 V (**c**) in 0.2 M N_2_-saturated PBS. Adapted from Guan et al. [90], Copyright 2018 Elsevier.

By 2018, Guan et al. [90] prepared a PtNi(3:1) alloy on N-doped carbon nano-fibres by electrospinning that exhibited an excellent sensitivity of 248.5 μA mM^−1^ cm^−2^ towards the reduction of H_2_O_2_ in an electrolyte of pH 7.24 standardised by PBS, reaching a relatively low limit of detection of 0.0375 μM. The studied nanocomposite alloy presented a high catalytic performance at a relatively low potential of −0.5 V vs. SCE, with the reduction current reaching −350 µA. The decay of the transient current, observed under a chronoamperometric analysis of the 1.0 mM H_2_O_2_ reduction reaction on PtNi/NCNFs/GCE, pointed to a diffusion-controlled process. This process allowed Cottrell’s expression to apply to obtain the catalytic rate constant for H_2_O_2_ reduction as 3.13 × 10^4^ mol^−1^ s^−1^. The resulting prepared electrode exhibited a progressive reduction current response with increasing the concentration of H_2_O_2_ (Figure 2a) in a rapid, sensitive (Figure 2b) and selective (Figure 2c) way.

As one of the most promising metal oxides in respect of its electrocatalytic performance toward H_2_O_2_ reduction, rare-earth oxides CeO_2_ grants a high electrical conductivity and the ability to mimic the activity of several natural redox enzymes. Although, the aggregation of CeO_2_-based nanocomposites declines their catalytic activity, limiting their efficient use. Considering this, Guan and coworkers [91] have developed a relevant procedure that enables the incorporation of CeO_2_ into nanocomposites through their anchoring on the surface of N-doped carbon nanofibers. Therefore, they have prepared a catalytic material that uniformly embeds the PtNi alloy/CeO_2_ plates onto high surface N-doped carbon nanofibers (PtNi/CeO_2_/NCNFs) with an exceptional sensitivity of 345.0 µA mM^−1^ cm^−2^ towards the cathodic reaction of H_2_O_2_. CV studies revealed that PtNi/CeO_2_/NCNFs electrode yielded a much higher reduction current density of −506.5 µA at a potential 0.1 V lower than the previously-synthesised PtNi/NCNFs/GCE [90]. The diffusion-controlled mechanism of the H_2_O_2_ reduction reaction was observed at the prepared PtNi/CeO_2_/NCNFs/GCE surface. The catalytic rate constant of the cathodic reaction of 1.0 mM H_2_O_2_ on the prepared electrode was as high as 3.52 × 10^4^ mol^−1^ s^−1^.

In the H_2_O_2_ electrochemical oxidation domain, Yang et al. [92] have prepared a porous structure of nanoparticles’ PtCu alloy that has an interconnected flower-like network skeleton that exhibits an outstanding detection performance towards H_2_O_2_ and glucose (GLU) electrochemical oxidation in near-neutral pH solutions. Particularly for H_2_O_2_ electrochemical oxidation, the prepared nanoporous material required a low working potential of 0.7 V vs. RHE and granted the detection of H_2_O_2_ in trace amounts as low as the detection limit of 0.1 μM. Over the range between 0.5 and 1.2 V, the PtCu alloy electrode onset potential is negatively shifted by more than 170 mV. It also reveals a superior catalytic activity for the electro-oxidation of H_2_O_2_ than Pt/C. The voltammetric behaviour of the PtCu alloy electrode towards the anodic reaction of the glucose has displayed a current density about 20 times higher than the obtained for the commercial Pt/C electrode.

Within the context of detecting endogenous H_2_O_2_, hybrid films based on bimetallic nanoparticles supported on reduced graphene sheets were prepared by Yu et al. [69] at various molar ratios of each metal. The sensor assembled by the prepared electrocatalyst has shown to be highly selective and sensitive to H_2_O_2_ electroreduction. Bimetallic Pt-Au/rGSs hybrid films have afforded the amperometric detection of H_2_O_2_ at a low potential of 0.0 V vs. SCE, with a detection limit of about 0.31 µM. All the anodic interferences resulting from electroactive species such as AA, UA, and dopamine (DA), as well as the cathodic interference from endogenous O_2_, were avoided at this low working potential. This type of metallic blend provides a comprehensive linear response to H_2_O_2_ and excellent selectivity. Therefore, the prepared sensor was successfully used to detect H_2_O_2_ released from PC12 pheochromocytoma cells and thus showed to be a promising alternative for in vivo H_2_O_2_ monitoring in the diagnostics domain [69].

In 2017, Hong Li et al. [93] developed a carbon fibre microelectrode (CFME) with a sensitive Pt-Pd alloy for H_2_O_2_ detection by electrochemical deposition. The microelectrode, whose nanocoral-shaped alloy possesses a high active surface favourable for a fast and efficient electron transfer, grants it a superior catalytic activity for H_2_O_2_ detection. Under ideal conditions, the prepared Pt-Pd/CFME microelectrode showed excellent response time (≤5 s), a high sensitivity (11,600 μA mM^−1^ cm^−2^), and a low detection limit (0.42 μM) under a wide linear range (5–3920 μM) for H_2_O_2_ detection. Furthermore, the prepared microelectrode was successfully used in a sensor to monitor H_2_O_2_ at the cellular level, namely from A549 epithelial cells and the amount of H_2_O_2_ in milk.

**Table 1:** Summary of main literature analytical parameters of various Pt-based alloy/bimetallic catalysts towards the electrochemical reduction of H_2_O_2_.

Electrocatalyst	Cyclic Voltammetry		Amperometry					Ref.
	E_pc_ (V)	I_pc_ (μA)	E_working_ (V)	*K*_cat_ (mol^−1^ cm^3^ s^−1^)	Linear Range (mM)	Sensitivity (μA mM^−1^ cm^−2^)	Detection Limit (μM)	
**SPGFE/Pt-PdBNC**	−0.05/Ag/AgCl	400.0 (10.0 mM H_2_O_2_)	−0.40		0.0050–6.00	804.0	0.870	[74]
**Fe@Pt/C**	−0.55/Ag/AgCl	300.0 (20.0 mM H_2_O_2_)	−0.40		0.0025–0.0416	218.9	0.750	[75]
**Pt_0.75_Ni_0.25_/C**	0.53/Ag/AgCl	1075.2 (30.0 mM H_2_O_2_)	−0.20	11.2 × 10^3^ (30.0 mM H_2_O_2_)				[18]
**Pt_0.75_Co_0.25_/C**	0.37/Ag/AgCl	1274.0 (30.0 mM H_2_O_2_)	−0.20	11.8 × 10^3^ (30.0 mM H_2_O_2_)				
**Pt-Sm**	−0.40/RHE	2025.0 (50.0 mM H_2_O_2_)	−0.40					[89]
**Pt/Fe_3_O_4_/rGO**	0.00/SCE	20.0 (0.2 mM H_2_O_2_)	0.00		0.1000–2.40	0.973	1.580	[79]
**PtNi/NCNFs(3:1)**	−0.50/SCE	350.6 (20.0 mM H_2_O_2_)	−0.10	31.3 × 10^3^ (1.0 mM H_2_O_2_)	0.0005–8.00	248.5	0.0375	[90]
**PtNi/CeO_2_/NCNFs**	−0.40/SCE	506.5 (20.0 mM H_2_O_2_)	−0.10	35.2 × 10^3^ (1.0 mM H_2_O_2_)	0.0005–15.00	345.0	0.025	[91]
**CNT/SiO_2_/(Au/Pt)**	−0.15/Ag/AgCl	23.0 (1.5 mM H_2_O_2_)	−0.10		0.0005–1.67		0.300	[82]
**Pt NF-N-Gr**	−0.40/Ag/AgCl		−0.40		0.0010–1.00	61.23	0.340	[80]
**Pt/PPy-C35%**	0.80/RHE	11,600.0 (50.0 mM H_2_O_2_)	0.90					[85]
**PtNi/MWCNTs**	−0.40/SCE	275.0 (10.0 mM H_2_O_2_)	−0.45		0.0002–24.60	2123.1	0.060	[87]
**Cu@Pt/C**	−0.30/SCE	310.0 (20.0 mM H_2_O_2_)	−0.30		0.0005–32.56	351.3	0.150	[68]
**np-PtCu**	0.30/RHE		0.70		0.0100–1.70	64.7	0.100	[92]
**PPy/Pt**	−0.175/Ag/AgCl	180.0 (4.0 mM H_2_O_2_)	−0.175		0.0250–0.50	305.45	0.600	[86]
**Cu@PtPd/C**	−0.10/Ag/AgCl		−0.10		0.0050–0.25	530.0	0.370	[88]
**Pt-Au/rGSs**	0.10/SCE	35.0 (0.1 mM H_2_O_2_)	0.00		0.0010–1.78	0.735	0.310	[69]
**Pt-Pd/CFME**	−0.50/Ag/AgCl	7.7 (5.0 mM H_2_O_2_)	−0.40		0.0050–3.92	11,600.0	0.420	[93]
**PtNPs@GR/GLN**	0.16/Ag/AgCl		0.14		0.00005–0.871	5643.0	0.037	[83]

Where *K*_cat_ is the rate constant of the cathodic reaction of H_2_O_2_ in each of the electrocatalysts used.

In addition to the easy release of competing analyte species from the active sites on the platinum surface, the extra atom in the alloy also changes the working potential of the nanocatalysts for H_2_O_2_ detection. Generally, the added atom in the platinum alloy interacts with platinum atoms, altering the electron energy levels in the alloy in such a way that the downshift in the energy of the substrate d-band centre changes the energy bond from the platinum d orbital. This way, it shifts the working potential towards potential values closer to zero, allowing the sensing of H_2_O_2_ at potentials where the coexisting species show a negligible electrochemical signal.

The optimised performance of some electrocatalysts allowed the selective detection of H_2_O_2_ among experimental samples having other concurrent bioactive species commonly present in biological fluid samples. For example, ascorbic acid (AA), uric acid (UA), dopamine (DA), glucose (GLU), and acetaminophen (AAP), which are regularly present in biological fluid samples, and other common metal ions such as NO_2_^−^, SO_4_^2−^, and Cl^−^ were added for measure along with the H_2_O_2_ [68,74,75,79,83,87,88,92,93]. The negligible current value verified for those electroactive species revealed an excellent selectivity of the reported electrodes towards the reduction of H_2_O_2_.

## 3. Electrocatalysts’ Intrinsic Catalytic Activity toward H_2_O_2_ Reduction

In the sector of the electrochemical detection of hydrogen peroxide, the main factor leading to the effective screening of low levels of H_2_O_2_ is the performance of the sensor’s transducer element. It corresponds to the electrode surface where the electrochemical reaction takes place.

Upon the catalytic action of an electrode, the H_2_O_2_ reduction reaction is likely to exhibit lower activation energy, thus improving its kinetics. Electrochemically, a lower overpotential at a specific current density should also be observed. As the most active precious metal against most chemical reactions in both acidic and alkaline electrolytes, platinum is the best known and most widely used catalyst. As a result of the d-orbital electron structure on the surface of its atoms, platinum induces a temporary binding of other molecules. Upon the adsorption of other molecules, the overall shape of the electronic cloud is rearranged so that the adsorbed molecules reassemble into new compounds. After the rearrangement, those molecules are driven off by other new inputs, but the electron structure of platinum as a catalyst does not change. Platinum’s low reactivity and valence band suitable for slow and weakly polar bonds with other atoms (mainly H and C) make its polycrystalline surface a good platform for the catalytic assignment of different reactions. As an excellent catalyst, platinum promotes the electrochemical reaction on its surface at a lower pressure and temperature in a shorter reaction time without being involved in it.

As mentioned above, despite its outstanding catalytic performance, platinum suffers from some significant drawbacks. One concerns the deactivation process of its catalytic activity by the adsorption of CO or some other reaction intermediates molecules to the Pt surface, blocking the active sites for heterogeneous catalysis [94]. Another drawback of bulk platinum as a catalyst material is its high cost. Platinum is scarce among precious metals. According to the market and consumption website Statista [95], the average worldwide platinum extraction represents only 170 metric tons in 2020, compared to other precious metals readily mined in more places such as gold (3030 tons) and silver (23,500 tons). Therefore, platinum trades at higher prices per unit based on its demands. In the prospect of a cost-effective electrode of comparable or superior catalytic performance, the construction of electrocatalysts by the fusion of various catalytic materials has been underway for half a century. The nature of the surface of bimetallic systems has been the subject of considerable research over the last forty years, considering these materials’ high catalytic activity and selectivity compared to their single metal counterparts [62].

Scientific findings related to Pt nanocomposite-based sensors for the sensitive detection of H_2_O_2_ highlight the enhanced catalytic effect achieved owing to the coexistence of other elements in the hybrid Pt composites. The improved catalytic performance of the Pt-based bimetallic alloys for H_2_O_2_ reduction is justified under two aspects:

1. Catalyst’ active surface area;
2. Synergistic effects of catalyst mixture.

### 3.1. Catalyst’ Active Surface Area

A bimetallic catalyst exhibits a larger active area, providing more active sites for breaking the molecular bonds and establish new bonds during catalysis than their single metallic counterparts. In practice, the reallocation of active sites on the surface of the electrocatalyst on account of its larger ECSA concerning its nominal area leads to an increase in catalytic activity.

Along with the extra atom in the platinum alloy, hybrid films with carbon allotropes provide extensive surface areas for uniform and homogeneous well-dispersed deposition of platinum nanoparticles. Moreover, including a generous and highly porous structure of carbon material in the Pt nanocomposite configuration will provide a larger adsorption area for the species that will undergo catalytic conversion. Such is the case for carbon allotropes of ordered mesoporous structure (Gr, rGS, and rGO) [69,79,80,83], organised carbon fibres [90,91,93], or even 1D planar structures of CNTs and MWCNTs [82,87].

In addition to mesoporous carbon structures, the high porosity silica (SiO_2_) also assembles a 3D support for the ultra-high density Au nanoparticles [82], expanding the extent of the ECSA for the catalytic reaction to occur, thus providing a sensitive response for the detection of H_2_O_2_ in neutral media (pH 7).

Considering the work of Morais et al. about Pt alloys electrocatalysts [18], it is possible to verify the effect resulting from the high distribution of active sites on the electrode surface. An increase in H_2_O_2_ reduction activity was observed in the Pt_0.75_M_0.25_/C alloys by experiencing a higher current density in an effective ECSA smaller than platinum itself. Indeed, the bimetallic cobalt alloy provides twice the current density at potential −0.2 V vs. SCE (19.0 mA cm^−2^ vs. 8.0 mA cm^−2^), with about half the specific surface area Pt/C (0.91 cm^2^ vs. 1.63 cm^2^). It leads to the appraisal of more free active sites on the surface of the Pt_0.75_Co_0.25_/C alloy for the H_2_O_2_ reduction reaction.

Accordingly, research on electrodes with highly irregular surfaces, either of high roughness [82,89] or high porosity [68,87], shows an increase in the electroactive surface available for heterogeneous catalysis of H_2_O_2_. The rough surfaces provide an additional number of active sites for the electrocatalytic reaction of the analyte, thus increasing the rate of the reduction reaction of the hydrogen peroxide.

### 3.2. Synergistic Effects of Catalyst-Blend Mixture

The interaction between the various metals comprising the alloy can effectively improve the electrocatalytic activity of the alloy. These bimetallic structures [96] are usually in the shape of core/end nanostructure alloys and mixed-metal nanoparticles. At this stage, the underlying mechanism and the factors that affect the catalytic properties of the bimetallic alloys owing to the addition of the second metal atom in the platinum atomic lattice are not entirely understood. However, some ideas have already been suggested to explain the improved catalytic activity attained by bimetallic catalysts [96]. These are based on the factors that provide a better interaction of the reactant and intermediate species to the catalyst’ active sites, i.e., on three different chemical effects that dictate the adsorption energies of the analyte species to the catalyst surface. They are divided into three categories; (i) the geometric factor, (ii) the electronic effect, and (iii) the co-catalytic effect:

(i). Geometric factor: A change in the surface geometry of the catalyst surface as a result of distance from its nearest neighbour atom (*strain* or *ensemble* effects);
(ii). Electronic effect: A change in the reactivity of the catalyst as a result of electron transfer or polarisation between the two adjacent metals, which leads to a change in the width of the surface d-band and a shift in the binding energy aimed at constant band filling (*ligand* or *electronic* effect);
(iii). Co-catalytic effect: Upon adding a second metal element in the Pt lattice, it can provide a reinforced adsorption site for some intermediate species or reactants, thereby enhancing their interaction with the catalyst. It is referred to as a *synergistic* effect because the combined action of both metals fosters improved adsorption to the catalyst. This way reinforces the catalytic ability of the bimetallic NPs.

As mentioned above in the nickel and cobalt alloys research [18], the kinetics of the electroreduction of H_2_O_2_ in polycrystalline Pt alloys are much more efficient in electrolytes free from other simultaneous highly adsorbing species [97]. Indeed, an enhancement of H_2_O_2_ reduction activity on Pt-alloys, Pt_0.75_M_0.25_/C, was observed due to the higher availability of the Pt active site on this kind of electrode surface.

**Figure 3:**
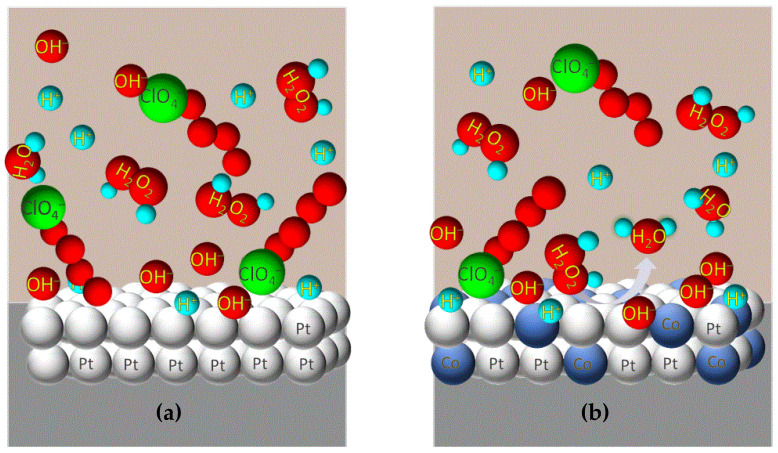
The catalysis of platinum on H_2_O_2_ reduction is constrained by the constant presence of adsorbed species on its surface (**a**). The presence of second metal in the electrode structure with the consequent alteration of the binding force of adsorbed species on the electrode surface results in a significant increase in active sites number for the catalysis of H_2_O_2_ cathodic reaction into H_2_O (**b**).

The catalytic performance of carbon-supported electrocatalysts for the cathodic reaction of H_2_O_2_ directly depends on the electrode reaction rate, which relies on the availability of Pt active sites [18]. H_2_O_2_ will have to “compete” with other co-adsorbed analyte species capturing the active sites on the Pt surface during its cathodic reaction (Figure 3a). This seizure has an inhibitory effect, delaying the catalytic activity for the cathodic response of H_2_O_2_. Thus, the presence of a second metal (similar to that portrayed in Figure 3b) will affect the electronic configuration of the outer Pt atoms at the electrode surface, so the bonds of the adsorbed species on the electrode surface are weakened, helping their removal upon their catalysis (*electronic* effect). It turns available the active Pt sites again for the catalysis of H_2_O_2_ reduction.

The surface structure of the electrode also influences the susceptibility to ion adsorption on the Pt surface. Therefore, the electrode’s charge and the fraction of adsorbed anions concomitantly will influence the reactivity of Pt alloy single crystals on the electrode for the H_2_O_2_ reduction reaction. This way, the undesired adsorption of the anions from the electrolyte solution onto the platinum electrode surface will also contribute to the delay in the kinetics of the H_2_O_2_ cathodic reaction.

Similarly, the susceptibility to ion adsorption on the surface of platinum atoms is influenced by the change it undergoes due to the proximity of electrons from neighbouring atoms. The acquisition of different spatial arrangements leads to the distortion of the fcc unit cell structure of crystalline Pt on the electrode surface. Thus, the ensemble effect on the adsorption energy of H_2_O_2_ on the electrode surface is also evident by the Pt alloys exhibiting a change in the adsorption of unwanted anions from the electrolyte solution.

Therefore, the electrode’s charge and the fraction of concurrently adsorbed anions will set Pt single crystals’ reactivity along with the second metal at the electrode for the H_2_O_2_ reduction reaction.

Similarly, the occurrence of a less electronegative atom in the vicinity of platinum, such as copper [68,87], iron [75], nickel [18,87,90,91], cobalt [18], and palladium [74,92,93] will create an inductive effect on Pt local electron density, by reducing its d-band electrons’ energy. Such an electronic properties shift reinforces the binding of the H_2_O_2_ and the intermediates OH_ads_ and H^+^ on the catalyst surface, improving the catalytic action of the electrode surface towards H_2_O_2_ sensing.

Considering that metallic nanoparticles’ support properties have an essential effect on the performance of the electrocatalysts used for the sensitive detection of H_2_O_2_, the carbon-based material as a support is a good choice as it provides good conductivity and a high ECSA and can stabilise the nanoparticles. However, they must also have good corrosion resistance in the reaction medium, which is not always the case.

In aqueous solutions, carbon support materials may experience some corrosion problems, mainly when used as a cathode in the sensing devices, whenever the electrode potential goes up to 1.5 V vs. the reversible hydrogen electrode [98]. Such decomposition, catalysed by the presence of Pt nanoparticles deposited onto the carbon support, is responsible for the loss of carbon material that proceeds through the evolution of CO_2_, which acts as a passive species for the cathode reaction. This deterioration of the carbon is associated with the aggregation of the Pt catalyst nanoparticles on the carbon surface as corrosion intensifies, resulting in a decrease in ECSA and consequent fall in electrocatalytic performance and lifespan of the Pt nanoparticle-based electrode.

Therefore, one of the most promising approaches is incorporating more resistant supporting materials into the catalyst nanostructure, such as conductive metal oxides and conjugated conducting polymers.

Metal oxide nanoparticles are nanomaterials that exhibit intrinsic enzyme activity [99], referred to as “nanoenzymes.” Though structurally different from natural enzymes (defined as three-dimensional structures of amino acid sequences), the crystal structures of metal oxide nanomaterials share certain similarities. These include their global size, shape, and surface charge, which allow them to mimic the action of natural enzymes. Indeed, iron oxide (Fe_3_O_4_) nanoparticles are one of the most typical nanozymes as they exhibit similar intrinsic enzymatic activity [100] to the biological horseradish peroxidase towards H_2_O_2_ reduction. Similarly, the high catalytic performance of cerium oxide (CeO_2_) is well known [99]. This catalytic activity stems from the mixed-valence states of the redox pair Ce^3+^ and Ce^4+^ that can easily switch between each state in a cyclic process and from the presence of vacant oxygen sites that stabilise the Ce^3+^ oxidation state and make the oxygen lattice highly flexible. Likewise, cerium oxide nanoparticles exhibit a catalytic activity mimetic of several natural enzymes, including catalase which catalyses the breakdown of hydrogen peroxide into molecular oxygen and water. Accordingly, the concerted action of these nanoenzymes along with the platinum-based nanocomposites may explain the improved catalytic activity of the electrodes of Pt/Fe_3_O_4_/rGO [79], PtNi/CeO_2_/NCNFs [91] hybrid films for the selective and sensitive detection of H_2_O_2_.

Conductive mesoporous type polymers have potential applications as catalyst supports. Particularly polypyrrole (PPy) is a promising support material due to its good electrical conductivity and high stability in electrochemical processes [101]. In addition to providing higher catalyst stability when used as a support component for carbon-based and metal nanoparticles, PPy used as a support material for metal nanoparticles provides an effective structure for the charge transfer on the electrode surface. It is expected that hybridised nanostructures of both conductive materials display a higher number of active sites and high electrical conductivity. Such hybridised nanostructure acts as high efficient co-catalysts (synergistic effect) in the H_2_O_2_ sensing [85,88].

## 4. Conclusions

The literature data reported in this review presented several proposals for coordinating Pt-based composites (in the form of alloys or single NPs) with other conductive materials, aiming to reduce the amount of platinum and simultaneously obtain a highly H_2_O_2_-sensitive transduction element. The literature surveys disclosed that catalysts of Pt-based alloys exhibit low working potential, fast electrochemical response, good reproducibility, and high sensitivity in the detection of H_2_O_2_.

The current study may provide a promising platform for non-enzymatic electrodes and affinity matrix assembly. The proposals presented for the alternative use of improved Pt-based alloy catalysts for the electro-reduction of H_2_O_2_ may be of practical interest for the assembly of sensors that can result in high-sensitive electrodes with lower Pt loadings.

Compared to the Pt-based catalyst, hybrid bimetallic catalysts often have a higher ECSA for electron transfer, which afford a higher catalytic activity. In addition, the increased free binding active sites of the Pt-based alloys electrocatalyst versus Pt have shown a higher ability to interact with the analyte, increasing the catalytic activity for H_2_O_2_ sensing. The hybrid electrocatalysts displayed good electrochemical performance for sensing H_2_O_2_ without any mediator or the addition of enzymes, showing a high peak current signal at a relatively low potential. The high performance of Pt-based bimetallic alloys for the sensing of H_2_O_2_ in a neutral media has also been observed. It is believed that these findings may guide the development of more efficient materials for highly sensitive electroanalysis of H_2_O_2_ for diagnostic purposes and therapeutic drug monitoring.
